# Country-level predictors of COVID-19 mortality

**DOI:** 10.1038/s41598-023-36449-x

**Published:** 2023-06-07

**Authors:** Paul A. Brown

**Affiliations:** Department of Basic Medical Sciences, Faculty of Medical Sciences Teaching and Research Complex, The University of the West Indies, Mona, Kingston 7, Jamaica

**Keywords:** Diseases, Risk factors

## Abstract

This study aimed to identify country-level predictors of COVID-19 mortality, after controlling for diverse potential factors, and utilizing current worldwide mortality data. COVID-19 deaths, as well as geographic, demographic, socioeconomic, healthcare, population health, and pandemic-related variables, were obtained for 152 countries. Continuous variables were examined with Spearman’s correlation, categorical variables with ANOVA or Welch’s Heteroscedastic F Test, and country-level independent predictors of COVID-19 mortality identified by weighted generalized additive models. This study identified independent mortality predictors in six limited models, comprising groups of related variables. However, in the full model, only WHO region, percent of population ≥ 65 years, Corruption Perception Index, hospital beds/100,000 population, and COVID-19 cases/100,000 population were predictive of mortality, with model accounting for 80.7% of variance. These findings suggest areas for focused intervention in the event of similar future public health emergencies, including prioritization of the elderly, optimizing healthcare capacity, and improving deficient health sector-related governance.

## Introduction

Coronavirus disease 2019 (COVID-19) is caused by severe acute respiratory syndrome coronavirus 2, an enveloped single-strand-RNA β-coronavirus, from the family *Coronaviridae*^1,2^. The virus was initially named 2019 novel coronavirus, after isolation from patients with viral pneumonia in Wuhan China in late December 2019^3^. Poor outcomes have been associated with multiple host factors, including age, sex, comorbidities, laboratory markers and lack of vaccination^1,4,5^. Higher mortality was also previously associated with the delta variant^6–8^. This variant originated in October 2021 in India and has three clades, 21A, 21I and 21J, with latter dominating across continents^9^. More recent work suggests delta variant was not more fatal than pre-delta variants^4^, but more fatal than omicron variant^5^.

However, World Health Organization describes a wide range of determinants of health, encompassing aspects of our social, economic, and physical environments, in addition to personal characteristics and behaviors^10^. It is therefore plausible that in addition to individual risks, country-level factors, including demographic-, socioeconomic-, and environment-related health parameters, may play an important role in COVID-19 incidence and subsequent mortality.

Previous authors have reported on COVID-19 mortality risk factors within geographic regions^11–14^, or during defined pandemic phases e.g., the first wave^12,14^. However, other authors have reported worldwide country-level COVID-19 mortality risk factors using data close to time of publication. Implicated risk factors include general pre-pandemic variables comprising country-level demographics (population ≥ 60 years^15^); socioeconomic and governance factors e.g., higher GDP per capita^16,17^, higher income disparity^16^, higher transport infrastructure quality and lower government effectiveness^18^; national healthcare metrics including lower general health expenditure, lower infectious disease system responsiveness and greater accountability^19^; and population health characteristics like higher prevalence of obesity^15,16^, chronic obstructive pulmonary disease, Alzheimer’s disease, and depression^17^. Reported risk factors also comprise pandemic-specific variables. These include pandemic features e.g., duration^15^ and case load^19^; pandemic-associated public health policies inclusive of delayed international travel restrictions^15^ and lower testing^18^; and pandemic-associated public behaviours, e.g., shorter duration of mask wearing^15^. In addition, prior work also explored worldwide country-level COVID-19 mortality with a focus on select predictors, for example health system parameters^19,20^.

However, to the best of my knowledge, no report has tested potential country-level predictors of COVID-19 mortality using a wide range of potential predictors, unrestricted geographic scope, and late 2022 COVID-19 mortality data. This study therefore aimed to identify country-level independent predictors of COVID-19 mortality after controlling for a diverse range of potential factors utilizing current worldwide mortality data.

## Results

### Study data

There were no duplicates or invalid entries. Number of countries with missing data ranged from 0 (Country) to 46 (Delta21Jp100K). One hundred and fifty-two countries had complete data for all variables. The study data therefore comprised 152 cases (countries) and 41 variables, used for all analyses.

Descriptive statistics are presented in Table 1. The dataset comprised 38, 26, 18, 48, 8, and 14 countries within the Africa, Americas, Eastern Mediterranean, Europe, South-East Asia, and Western Pacific UN-regions, respectively. COVID-19 deaths/100,000 population ranged widely between 0.13 and 660.38. Cases/100,000 population also ranged widely from 39.12 to 70,445.77. Mean per capita deaths/cases were 26.7/2300, 204/13,531, 73.3/9334, 251/33,367, 47/6971 and 44.2/18,842, within the Africa, Americas, Eastern Mediterranean, Europe, South-East Asia, and Western Pacific UN-regions, respectively, with Africa reporting the lowest per capita cases and deaths.

**Table 1:** Descriptive statistics.

Variable	Minimum	Mean	Median	Maximum	SD	IQR	Skewness	Kurtosis
CovDp100K	0.13	135.96	92.15	660.38	138.21	194.97	1.18	1.03
AvgTemp	− 3.71	19.01	21.74	30.01	8.24	14.09	− 0.63	− 0.69
pPopFem_Yr	2021.00	2021.00	2021.00	2021.00				
pPopFem	25.00	49.77	50.30	53.90	3.71	1.20	− 4.23	20.78
pPop65_Yr	2021.00	2021.00	2021.00	2021.00				
pPop65	1.00	9.95	7.50	29.00	6.96	12.00	0.61	− 0.99
pPopUrb_Yr	2021.00	2021.00	2021.00	2021.00				
pPopUrb	13.00	62.40	64.00	100.00	21.83	35.00	− 0.35	− 0.76
AdLit_Yr	2021.00	2021.00	2021.00	2021.00				
AdLit	0.00	82.70	93.65	100.00	23.28	25.08	− 1.59	1.69
GDPpC_Yr	2014.00	2020.91	2021.00	2021.00				
GDPpC	236.80	16,628.54	6312.80	135,682.80	23,124.36	18,265.03	2.27	5.90
IGSpGDP_Yr	2004.00	2020.39	2021.00	2021.00				
IGSpGDP	1.90	45.28	40.15	176.70	25.44	27.97	2.00	6.47
pWfUem_Yr	2021.00	2021.00	2021.00	2021.00				
pWfUem	0.30	8.36	6.40	33.60	6.07	6.35	1.52	2.45
CPI_Yr	2021.00	2021.00	2021.00	2021.00				
CPI	14.00	44.34	40.00	88.00	18.63	26.25	0.68	− 0.40
HEpGDP_Yr	2011.00	2018.89	2019.00	2019.00				
HEpGDP	1.80	6.37	6.20	16.77	2.58	3.88	0.61	0.54
GHS.In_Yr	2021.00	2021.00	2021.00	2021.00				
GHS.In	16.70	41.79	39.35	75.90	13.65	22.50	0.43	− 0.78
LEB_Yr	2020.00	2020.00	2020.00	2020.00				
LEB	54.00	72.93	74.00	85.00	7.43	10.25	− 0.63	− 0.38
HBp100K_Yr	2021.00	2021.00	2021.00	2021.00				
HBp100K	0.00	19.93	14.60	94.00	17.30	21.42	1.61	3.35
MDp100K_Yr	2021.00	2021.00	2021.00	2021.00				
MDp100K	0.10	22.91	22.40	84.50	18.96	30.97	0.62	− 0.26
NCDMortp100K_Yr	2021.00	2021.00	2021.00	2021.00				
NCDMortp100K	13.80	71.50	69.60	100.00	16.48	22.25	− 0.42	0.26
pPopH20_Yr	2007.00	2019.86	2020.00	2020.00				
pPopH20	37.00	89.01	97.00	100.00	15.62	15.00	− 1.55	1.31
PoeMgt_Yr	2021.00	2021.00	2021.00	2021.00				
CovCp100K	39.12	16,634.37	9672.71	70,445.77	18,106.17	25,029.41	1.07	0.05
Delta21Jp100K	0.00	44.73	13.85	927.08	93.53	34.53	6.18	52.87
VacFullp100	0.23	53.56	57.04	99.01	25.06	41.14	− 0.33	− 1.03

AdLit = adult literacy rate (percent of 15+ years); AvgTemp = average temperature (2021); CovCp100K = COVID-19 Cases/100,000 population; CovDp100K = COVID-19 Deaths/100,000 population; CPI = Corruption Perception Index; Delta21Jp100K = COVID-19 Delta 21J sequence count/100,000 tests; GDP = gross domestic product; GDPpC = GDP per capita (US$); GHS.In = Global Health Security Index; HBp100K = hospital beds/100,000 population; HEpGDP = health expenditure (% of GDP); IGSpGDP = imports of goods and services (% of GDP); IQR = interquartile range; LEB = life expectancy at birth; MDp100K = doctors/100,000 population; NCDMortp100K = non-communicable disease (age-standardized) mortality rate/100,000 population; pPop65 =  ≥ 65 years of age (percent of population); pPopFem = females (percent of population); pPopH20 = using at least basic drinking water services (percent of population); pPopUrb = urban population (percent of population); pWfUem = unemployment (percent of workforce); VacFullp100 = number of persons fully vaccinated/100 population; SD = standard deviation; Yr = year of latest available data for the associated variable.

Data quality checks revealed most variables contained recent data (2018–2022), as seen with GDPpC_Yr, IGSpGDP_Yr, HEpGDP_Yr, and pPopH20_Yr, with minimum year of 2014, 2004, 2011, and 2007 respectively. Datapoints earlier than 2018 occurred in 1, 5, 2, and 3 cases for GDPpC, IGSpGDP, HEpGDP, and pPopH20, respectively. Thirteen variables contained outliers, but these were relatively few for most variables. Quality checks also identified a few additional datapoints of potential concern, including within the AdLit and HBp100K variables, with a solitary minimal value of 0, for Chad and Mali, respectively. However, these were not outliers as confirmed with the *boxplot* function (*graphics* package^21^), and therefore not removed. Also, no outliers were removed as they were judged to represent valid data after visualization with *plot* function (*base* package^22^).

### Correlation analysis

Correlations between COVID-19 mortality and continuous variables of interest ranged between − 0.58 and 0.78, with all 19 correlations being statistically significant, and the strongest correlation being with COVID-19 cases per capita (Table 2). Among the 19 associations, there were 1, 7, and 10 small, medium, and large positive correlations, as well as 1 large negative correlation (Table 2, Supplementary Fig. S1).

**Table 2:** Summary of correlation analysis.

Dependent	Independent	rho^a^	95% CI	Effect size^b^	p (2-sided)
CovDp100K	AvgTemp	− 0.58	[− 0.68, − 0.46]	Large	< 0.001
CovDp100K	pPopFem	0.42	[0.27, 0.54]	Medium	< 0.001
CovDp100K	pPop65	0.77	[0.69, 0.83]	Large	< 0.001
CovDp100K	pPopUrb	0.43	[0.28, 0.55]	Medium	< 0.001
CovDp100K	AdLit	0.67	[0.57, 0.75]	Large	< 0.001
CovDp100K	GDPpC	0.63	[0.52, 0.72]	Large	< 0.001
CovDp100K	IGSpGDP	0.32	[0.17, 0.46]	Medium	< 0.001
CovDp100K	pWfUem	0.30	[0.15, 0.45]	Medium	< 0.001
CovDp100K	CPI	0.49	[0.36, 0.61]	Medium	< 0.001
CovDp100K	HEpGDP	0.58	[0.47, 0.68]	Large	< 0.001
CovDp100K	GHS.In	0.63	[0.51, 0.72]	Large	< 0.001
CovDp100K	LEB	0.60	[0.49, 0.70]	Large	< 0.001
CovDp100K	HBp100K	0.63	[0.53, 0.72]	Large	< 0.001
CovDp100K	MDp100K	0.73	[0.65, 0.80]	Large	< 0.001
CovDp100K	NCDMortp100K	0.44	[0.30, 0.56]	Medium	< 0.001
CovDp100K	pPopH20	0.66	[0.56, 0.75]	Large	< 0.001
CovDp100K	CovCp100K	0.78	[0.70, 0.84]	Large	< 0.001
CovDp100K	Delta21Jp100K	0.25	[0.09, 0.40]	Small	0.002
CovDp100K	VacFullp100	0.37	[0.22, 0.50]	Medium	< 0.001

AdLit = adult literacy rate (percent of 15+ years); AvgTemp = average temperature (2021); CI = confidence interval; CovCp100K = COVID-19 Cases/100,000 population; CovDp100K = COVID-19 Deaths/100,000 population; CPI = Corruption Perception Index; Delta21Jp100K = COVID-19 Delta 21J sequence count/100,000 tests; GDP = gross domestic product; GDPpC = GDP per capita (US$); GHS.In = Global Health Security Index; HBp100K = hospital beds/100,000 population; HEpGDP = health expenditure (% of GDP); IGSpGDP = imports of goods and services (% of GDP); LEB = life expectancy at birth; MDp100K = doctors/100,000 population; NCDMortp100K = non-communicable disease (age-standardized) mortality rate/100,000 population; pPop65 =  ≥ 65 years of age (percent of population); pPopFem = females (percent of population); pPopH20 = using at least basic drinking water services (percent of population); pPopUrb = urban population (percent of population); pWfUem = unemployment (percent of workforce); VacFullp100 = number of persons fully vaccinated/100 population.

^a^Spearman’s correlation coefficient (two-sided), degrees of freedom = 150.

^b^Based on Cohen’s criteria^23^.

### ANOVA analysis/Welch’s Heteroscedastic F Test

Summary of the ANOVA and Welch’s Heteroscedastic F Test results are presented in Table 3. Mean COVID-19 deaths did not differ across PoeMgt groups. However, there was a statistically significant difference across WHO_Region groups. Pairwise comparisons demonstrated significant differences (two-sided) between several pairs (Supplementary Table S1).

**Table 3:** Summary of ANOVA/Welch’s Heteroscedastic F Test.

ANOVA						
	df	Sum Sq	Mean Sq	F	p (1-sided)	R^2^
PoeMgt	2	61,628	30,814	1.626	0.2	0.021
Residuals	149	2,822,852	18,945			

Welch’s Heteroscedastic F Test						
	df_num_	df_denom_	Statistic	p (1-sided)		
WHO_Region	5	51.26133	29.86029	< 0.001		

df = degrees of freedom; denom = denominator; F = F statistic; Mean Sq = mean square; num = numerator; PoeMgt = point of entry management; Sum Sq = sum of squares; WHO_Region = WHO region.

### Multivariate analysis

The GAMs analyzed included 6 limited models (geographic, demographic, socioeconomic, health metric, population health, and pandemic-related) and one full model. As shown in Table 4, WHO_Region and s(AvgTemp), s(pPop65) and s(AdLit), s(pWfUem) and s(CPI), s(HEpGDP) and s(MDp100K), s(pPopH20), and s(CovCp100K), were identified as independent predictors of COVID-19 mortality for the gam.MODEL_1Geo, gam.MODEL_2Dem, gam.MODEL_3SoEc_mod, gam.MODEL_4Heal_mod, gam.MODEL_5PopH, and gam.MODEL_6Pand models respectively. For the gam.MODEL_Full_mod model, independent predictors were WHO_Region, s(pPop65), s(CPI), s(HBp100K), and s(CovCp100K), accounting for 80.7% of variance. All models demonstrated practical significance with at least medium effect size: R^2^ > 13% or Cohen’s f^2^ > 0.15^23^. Models fitted with standardized independent variables produced similar results. Backward elimination using the R^2^ criterion, identified gam.MODEL_Full_mod as most parsimonious, without further modification (not shown).

**Table 4:** Independent predictors of COVID-19 mortality.

Model/variable^a^	Coefficient^c^	EDF	t/F	p-value^d^	R^2^ (%)^e^	f^2f^	Power^f^
gam.MODEL_1Geo					58.7	0.956	1.00
Intercept	33.906	1.000	4.355	0.000			
WHO_Region: Americas	162.379	1.000	7.719	**0.000**			
WHO_Region: Eastern Mediterranean	43.162	1.000	3.287	**0.001**			
WHO_Region: Europe	168.861	1.000	7.599	**0.000**			
WHO_Region: South-East Asia	27.732	1.000	1.698	0.092			
WHO_Region: Western Pacific	22.890	1.000	1.964	0.051			
s(AvgTemp)	34.646	2.142	5.033	**0.004**			
gam.MODEL_2Dem					60.4	0.926	1.00
Intercept	113.384	1.000	17.250	0.000			
s(pPopFem)	− 10.148	1.810	1.371	0.289			
s(pPop65)	120.969	2.064	14.395	**0.000**			
s(pPopUrb)	18.034	2.286	1.860	0.243			
s(AdLit)	− 4.664	2.794	6.911	**0.000**			
gam.MODEL_3SoEc_mod^b^					30.7	0.325	1.00
Intercept	116.803	1.000	12.500	0.000			
s(pWfUem)	45.540	1.415	3.338	**0.024**			
s(CPI)	79.084	1.492	20.556	**0.000**			
gam.MODEL_4Heal_mod^b^					53.6	0.935	1.00
Intercept	119.299	1.000	16.980	0.000			
s(HEpGDP)	30.374	1.892	8.218	**0.000**			
s(LEB)	65.371	2.164	3.775	0.057			
s(HBp100K)	79.553	1.911	2.253	0.063			
s(MDp100K)	53.354	1.448	5.084	**0.014**			
gam.MODEL_5PopH					45.5	0.373	1.00
Intercept	108.766	1.000	12.370	0.000			
s(NCDMortp100K)	16.963	2.408	0.477	0.699			
s(pPopH20)	− 25.640	2.419	23.591	**0.000**			
gam.MODEL_6Pand					61.3	0.603	1.00
Intercept	103.031	1.000	11.180	0.000			
PoeMgt: future emergency plan	21.546	1.000	1.267	0.207			
PoeMgt: none	4.902	1.000	0.643	0.521			
s(CovCp100K)	215.521	1.526	70.287	**0.000**			
s(Delta21Jp100K)	6.918	1.104	0.726	0.434			
s(VacFullp100)	17.292	2.315	2.091	0.097			
gam.MODEL_Full_mod^b^					80.7	2.706	1.00
Intercept	101.832	1.000	5.902	0.000			
WHO_Region: Americas	66.142	1.000	2.849	**0.005**			
WHO_Region: Eastern Mediterranean	36.547	1.000	1.809	0.073			
WHO_Region: Europe	35.363	1.000	1.229	0.221			
WHO_Region: South-East Asia	5.706	1.000	0.207	0.836			
WHO_Region: Western Pacific	− 14.271	1.000	− 0.619	0.537			
PoeMgt: Future Emergency Plan	− 8.947	1.000	− 0.511	0.610			
PoeMgt: None	− 3.372	1.000	− 0.287	0.774			
s(AvgTemp)	27.531	2.569	1.141	0.303			
s(pPop65)	45.536	2.349	8.569	**0.000**			
s(pPopUrb)	8.429	2.543	0.538	0.675			
s(AdLit)	− 44.298	2.900	1.191	0.290			
s(pWfUem)	− 2.551	2.137	0.480	0.750			
s(CPI)	41.194	2.269	5.307	**0.002**			
s(HEpGDP)	− 8.210	2.514	0.544	0.642			
s(LEB)	63.659	2.535	2.618	0.056			
s(HBp100K)	11.532	2.324	3.344	**0.015**			
s(CovCp100K)	188.652	2.026	10.701	**0.000**			
s(Delta21Jp100K)	14.773	1.342	3.644	0.059			
s(VacFullp100)	21.700	2.479	0.432	0.725			

^a^All models with CovDp100K as dependent variable.

^b^Model modified by removing variable(s) with high partial concurvity.

^c^Coefficient for smooth terms = sum of basis function coefficients.

^d^p-values are approximations computed from relevant statistic and effective (estimated^24^) degrees of freedom^25^.

^e^Reported as “Deviance explained”^25^.

^f^f^2^ and power (rounded) computed with pwr.f2.test function (pwr package^26^).

1Geo = geographic; 2Dem = demographic; 3SoEc = socioeconomic; 4Heal = health metric; 5PopH = population health; 6Pand = pandemic-related; AdLit = adult literacy rate (percent ≥ 15 years^27^); AvgTemp = average temperature (2021); CovCp100K = COVID-19 Cases/100,000 population; CPI = Corruption Perception Index (0–100, where 100 = best^27^); Delta21Jp100K = COVID-19 Delta 21J sequence count/100,000 tests; EDF = effective (estimated^24^) degrees of freedom, a measure of smooth complexity, with 1 implying linearity^28^; F = F statistic (numeric variables); gam.MODEL = generalized additive model; GDP = gross domestic product; HBp100K = hospital beds/100,000 population; HEpGDP = health expenditure (% of GDP); LEB = life expectancy at birth; MDp100K = doctors/100,000 population; mod = modified; NCDMortp100K = non-communicable disease (age-standardized) mortality rate/100,000 population; PoeMgt = point of entry management; pPop65 =  ≥ 65 years of age (percent of population); pPopFem = females (percent of population); pPopH_2_0 = using at least basic drinking water services (percent of population); pPopUrb = urban population (percent of population); pWfUem = unemployment (percent of workforce); t = t statistic (intercept and categorical variables); VacFullp100 = number of persons fully vaccinated/100 population; WHO = World Health Organization; WHO_Region = WHO region.

Significant values are in bold.

Mortality varied across regions with highest mean deaths in Europe and the Americas. Initial analysis revealed that mortality was significantly greater in the Americas compared with Africa, with an average increase of 66.1 deaths/100,000 population. This analysis also suggested that the Americas and to a lesser extent Eastern Mediterranean and Europe appeared to stand out from the other regions. Additional models were therefore run with these WHO regions as reference. With the Americas as reference group, in addition to Africa, mortality was significantly greater in the Americas compared with South-East Asia and Western Pacific, with an average increase of 60.4 and 80.4 deaths/100,000 population respectively. With Eastern Mediterranean as reference group, mortality was significantly greater in this region compared with Western Pacific, with an average increase of 50.8 deaths/100,000 population. Finally, with Europe as reference group, mortality was significantly greater in Europe compared with Western Pacific, with an average increase of 49.6 deaths/100,000 population.

### Partial effects

Partial effects plots assessed impact of individual predictors. The gam.MODEL_1Geo model demonstrated greatest COVID-19 mortality at average temperature between approximately 10 °C to 15 °C (Supplementary Fig. S2). In the gam.MODEL_2Dem model, mortality increased with percent of population ≥ 65 up to approximately 20% then tapered off, but was initially unchanged with increasingly percent of adult literacy, before increasing above approximately 70% (Supplementary Fig. S3). Among socioeconomic variables, deaths increased logarithmically with Corruption Perception Index (CPI), but increased with greater percent unemployment up to approximately 15% before leveling off (Supplementary Fig. S4). COVID-19 death increased progressively with health expenditure relative to GDP and doctors/100,000, as shown in gam.MODEL_4Heal_mod model (Supplementary Fig. S5). In the gam.MODEL_5PopH and gam.MODEL_6Pand models, deaths increased exponentially with percent of population using at least basic drinking water services (Supplementary Fig. S6), but increased logarithmically with COVID-19 Cases/100,000 population (Supplementary Fig. S7), respectively.

**Figure 1:**
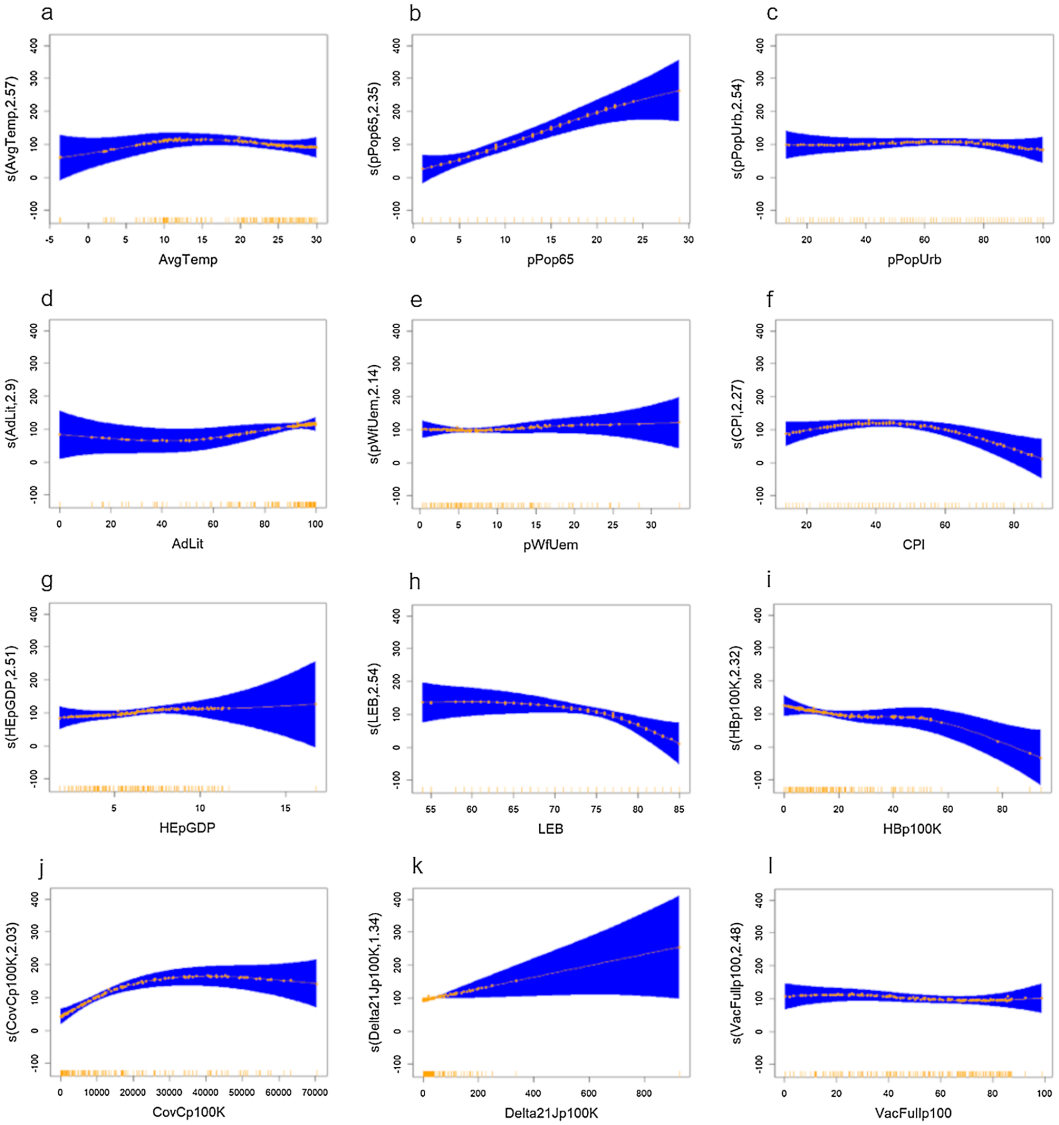
Partial effects plots for gam.MODEL_Full_mod model. (**a**) AvgTemp, (**b**) pPop65, (**c**) pPopUrb, (**d**) AdLit, (**e**) pWfUem, (**f**) CPI, (**g**) HEpGDP, (**h**) LEB, (**i**) HBp100K, (**j**) CovCp100K, (**k**) Delta21Jp100K, and (**l**) VacFullp100. Plotted with the *shift* argument to shift the scale based on the intercept value, for a more natural interpretation^28^. The smooth function on y-axis therefore represents the partial effect of the independent variable on COVID-19 mortality (presented as the independent variable, with effective degrees of freedom). Shading reflects 95% confidence interval for the mean shape of the effect^28^. AvgTemp = average temperature (2021); pPop65 =  ≥ 65 years of age (percent of population); pPopUrb = urban population (percent of population); AdLit = adult literacy rate (percent ≥ 15 years); pWfUem = unemployment (percent of workforce); CPI = Corruption Perception Index; HEpGDP = health expenditure (% of GDP); LEB = life expectancy at birth; HBp100K = hospital beds/100,000 population; CovCp100K = COVID-19 Cases/100,000 population; Delta21Jp100K = COVID-19 Delta 21J sequence count/100,000 tests; VacFullp100 = number of persons fully vaccinated/100 population.

Including all eligible variables in the gam.MODEL_Full_mod model, demonstrated that COVID-19 deaths increased progressively with greater percent population ≥ 65 and up to approximately 30,000 cases/100,000, but decreased progressively above a CPI of approximately 50 and above approximately 50 hospital beds/100,000 population. Assessment of the trend changes for these independent predictors comparing the modified full model with the corresponding smooth term versus the linear term, using the F-test^25^, demonstrated significant differences (1-sided) for CPI (F = 3.3735, p = 0.03838) and CovCp100K (F = 5.7823, p = 0.003924). The partial effects plots also suggested deaths increased progressively with increasing COVID-19 Delta 21J sequence count/100,000 tests, and decreased progressively above life expectancy at birth (LEB) of approximately 75. However, these latter trends were not statistically significant (Fig. 1).

### Differences in continuous independent predictors across who regions

As with COVID-19 deaths/100,000 population, WHO regions also differed significantly in percent of population ≥ 65 years of age (Welch's Heteroscedastic F Test statistic = 71.86161, df_num_ = 5, df_denom_ = 38.79369, p < 0.001), CPI (Welch's Heteroscedastic F Test statistic = 11.31819, df_num_ = 5, df_denom_ = 45.55676, p < 0.001), HBp100K (Welch's Heteroscedastic F Test statistic = 26.15499, df_num_ = 5, df_denom_ = 40.21732, p < 0.001), and COVID-19 cases/100,000 population (Welch's Heteroscedastic F Test statistic = 28.97315, df_num_ = 5, df_denom_ = 37.22894, p < 0.001). For all five variables tested across WHO regions, Africa had the lowest mean, while Europe had the highest. Pairwise comparisons demonstrated significant differences (two-sided) between several WHO region pairs for these variables: always including Africa vs. Europe and South-East Asia vs. Europe, as well as Africa vs. Americas for cases, deaths, and percent of population ≥ 65 years (Supplementary Tables  S1–S5).

### Linear modelling

Multiple linear regression identified similar independent predictors for the limited models, except the MODEL_1Geo model where only WHO_Region was predictive. However, in the corresponding full linear model (MODEL_Full_mod), COVID-19 mortality independent predictors were WHO_Region, pPop65, CPI, and AdLit, accounting for 63.2% of variance. Neither HBp100K nor CovCp100K were predictive (not shown).

## Discussion

This study identified WHO region, percent of population ≥ 65 years, CPI, hospital beds/100,000 population, and COVID-19 Cases/100,000 population as independent predictors of COVID-19 mortality, accounting for 80.7% of variance. Mortality varied across regions with highest mean deaths in Europe and the Americas. The partial effects plots further demonstrated that COVID-19 deaths increased progressively with greater percent of population ≥ 65 and up to approximately 30,000 cases/100,000, but decreased progressively above a CPI of approximately 50 and above approximately 50 hospital beds/100,000 population.

Mortality was significantly greater in the Americas, compared with Africa. Caseload was lowest for Africa in the dataset and consistent with WHO’s confirmation of under-reporting in this region^29^. However, the low African mortality was determined after controlling for eligible variables including caseload. Potential contributors to the low African mortality include younger age structure with associated reduced comorbidities, genetic factors including decreased response to angiotensin-converting enzyme inhibitors, natural selection conferring protection, trained immunity-based herd immunity, lower life expectancy, and low seeding rate due to lower air traffic to the continent^30–33^. Additional analyses revealed that mortality was also significantly greater in the Americas compared with South-East Asia and Western Pacific, in Eastern Mediterranean compared with Western Pacific, and in Europe compared with Western Pacific region. Various factors have been suggested for the low Western Pacific mortality including prior investment in pandemic preparation, as well as rapid and stringent public health responses, such as aggressive testing and early case management^34,35^. The causes of the lower Africa, South-East Asia, and Western Pacific mortality warrant further study.

Progressively greater COVID-19 mortality with increasing percent of population ≥ 65 is not surprising given previous similar findings for proportion of population ≥ 60^15^, and can be explained by multiple factors. These include atypical presentation of respiratory infections with associated delayed intervention and polypharmacy, age-related altered immune response, increased presence of multiple comorbidities, and polypharmacy-associated enhanced susceptibility to viral infections^36^. This finding is also consistent with the younger age-structure in Africa, contrasting that in Europe and the Americas. The initial enhanced mortality with increasing caseload also appears logical. It is less clear why mortality levels off beyond approximately 30,000 cases/100,000. Possible explanations include increasing competence^37,38^, or resource allocation^39^, in settings with high caseloads, offsetting the heightened burden.

There was progressively decreasing mortality above a CPI of approximately 50. CPI is a composite index derived from studies and expert surveys, published annually by Transparency International, and measures perceived public sector corruption. The index ranges from 1 to 100, with 100 indicating the lowest level of perceived corruption^40^, and is strongly correlated with other measures of corruption^41^. It is possible that higher levels of corruption could negatively impact reporting, and recent work has shown that high CPI was associated with increased daily reported COVID-19 cases and deaths. However, this analysis was restricted to data for the initial 120 days from first confirmed case^42^, and could be reflective of the early pandemic response. In contrast, the present CPI finding is consistent with findings of a significant negative association between CPI and poor health outcomes, and a positive association between health-sector corruption specifically and chronic disease, using data covering the period 2004–2015^43^. It is also consistent with evidence corruption undermines various aspects of healthcare system performance, including efficiency^44,45^. However, efficiency is not guaranteed by abundance of healthcare system inputs including health expenditure^46^, which may help explain why countries with comparable health expenditure differ with respect to important health outcomes including LEB and infant mortality rate^47^. This lack of congruence is consistent with the present finding that health expenditure was not an independent predictor of COVID-19 mortality in the modified full model.

The decreased mortality above approximately 50 hospital beds/100,000 population is also not consistent with some prior reports. An early study, using global October 2020 data, found no significant association between beds/100,000 and COVID-19 deaths^20^, a result that could at least partly be due to the early data. A more recent study found increased mortality in Italian regions with higher beds per capita, after adjusting for percentage of population ≥ 65/LEB/aging index, health expenditure per capita, general practitioners per 1000, and number of long-term care facilities^48^. However, as these authors suggest, regions with higher beds per capita are more centralized, and will likely attract higher caseloads and hence mortality. This is supported by the observation that hospital beds/100,000 population was lost as an independent predictor if percentage of population ≥ 65 and cases per capita were removed from the current paper’s modified full model. The current findings are also consistent with a USA report that regions with more general medicine/surgical beds per COVID-19 case had significantly lower COVID-19 mortality^39^. Populations served by less than 50 hospital beds/100,000 may therefore be at risk.

The study results suggest some important implications. They highlight the complex nature of the relationships under investigation. For example, even though average temperature, adult literacy rate, health expenditure, doctors per capita, and percent of population using at least basic drinking water services, were all highly correlated with mortality and identified as independent predictors in their respective limited models, these relationships were lost after controlling for all eligible variables in the full model. Likewise, the present study found that Africa had the lowest cases, deaths, and CPI among WHO regions. Low African caseload was consistent with the low mortality seen in both the relevant limited model and the full model. However, although there was a positive medium correlation between CPI and mortality and progressively greater mortality with increasing CPI in the relevant limited model, after adjusting for other variables in the full model, low CPI was associated with high COVID-19 mortality. This implies the low African CPI does not adequately explain the low COVID-19 mortality seen in Africa. Also, full vaccination per capita, moderately correlated with COVID-19 deaths, was not a predictor of mortality in the full model. Previous USA^49^ and global^50^ analyses suggested that vaccination reduces mortality. However, the USA analysis considered any level of vaccination, controlled for county population size, social vulnerability index, and mobility changes, and assessed data from the alpha/delta phase of the pandemic^49^, during which vaccinations may have been more impactful, considering the lower post-delta mortality risk^5^. Similarly, although the global analysis included a diverse range of covariates, the data was from late 2021/early 2022^50^, in proximity to the delta wave^4^. Therefore, the timing of the current dataset may partly explain why Delta 21J sequence count/100,000 tests was not identified as an independent predictor of COVID-19 mortality. These findings suggest the need for future work to determine the temporal relationships between COVID-19 mortality and potential predictors. The results also imply that a substantial portion of COVID-19 mortality risk originates from factors beyond the control of individuals. Accordingly, the WHO’s Sustainable Development Goal 3, “Good Health and Well-Being”^51^, arguably represents a justifiable mandate for countries to assume substantial responsible for the welfare of their citizens. However, further work also seems prudent to assess the impact of other potential predictors relevant to personal responsibility.

Among the study’s strengths, the dataset comprised complete data on 152 countries, with representation from all UN-defined regions. A comprehensive list of variables was also included in the models. This was important, as reported COVID-19 deaths may also depend on extraneous factors including population demographics, governance, and health system capacity. Including such variables therefore facilitated controlling for diverse factors. Additionally, the methodology utilized generalized additive models, allowing for analysis and visualization of complex, non-linear relationships. Regarding analysis, use of GAMs probably explains why HBp100K and CovCp100K were identified as independent predictors in the gam.MODEL_Full_mod model, but not the linear MODEL_Full_mod model, based on their clearly non-linear partial effects plots. Regarding visualization, comparing gam.MODEL_Full_mod with corresponding smooth term versus linear term for CPI and CovCp100K, demonstrated significant differences, implying non-linear trends, supporting utility of visualizing trend changes with GAM-based partial effects plots.

There were also some limitations. Firstly, the cross-sectional design prevents causal assumptions. In addition, the database used was dependent on available sources. It is possible that some sources under-reported COVID-19 cases and deaths, as suggested for Africa^29^, as well as other variables. The results must therefore be interpreted accordingly. Further, data quality could have varied between different sources. However, the overall data quality was generally good, with most data being recent (2018–2022), with only a solitary datapoint in two variables appearing potentially questionable.

In conclusion, COVID-19 mortality varied across regions with highest mean deaths in Europe and the Americas. Mortality increased progressively with increasing population ≥ 65, as well as with caseload up to ~ 30,000/100,000 population. Finally, mortality decreased progressively at high CPI and high hospital beds per capita. These findings suggest areas for focused intervention in the event of similar future public health emergencies, including prioritization of the elderly, optimizing healthcare capacity, and improving deficient health sector-related governance.

## Methods

### Study design and sample size calculation

This was a cross-sectional study. The required sample size was calculated based on the Raosoft online sample size calculator at http://www.raosoft.com/samplesize.html. Assuming a population of approximately 200 United Nation-defined countries, the minimum required sample size to achieve a 5% margin of error at the 95% confidence level was 132 countries.

### Raw data

Data was obtained from several sources (Table 5), including two datasets from the World Health Organization (WHO); one from Trading Economics; nine from the World Bank; and one each from the Nuclear Threat Initiative/Johns Hopkins Center for Health Security/Economist Impact (NTI/JHCHS/EI), CoVariants, and Worldometer. Country, WHO region, as well as COVID-19 cases and deaths were from 16th December 2022. COVID-19 Delta 21J sequence count per country (based on data made available by GISAID: the Global initiative on sharing all influenza data^52,53^) was from 15th December 2022. The per capita vaccination data was from 13th December 2022, and average temperature data was for 2021. Other variables were obtained for the most recent year for each country. As a result, for each of these variables, the year of collection varied between countries, which was collected as an associated “_Yr” variable (Table 1). All variables were numeric, except for WHO region and point of entry management (PoeMgt). There were six WHO regions: Africa, Americas, Eastern Mediterranean, Europe, South-East Asia, and Western Pacific. PoeMgt was measured with 3 groups: no plan; plan between public health system and border control authorities to identify international cases, and trace and quarantine contacts in response to active public health emergencies; and plan between public health system and border control authorities to identify international cases, and trace and quarantine contacts to prepare for future public health emergencies^27^. All variables were used as obtained from source, except for Delta 21J sequence count/100,000 tests, which was computed based on sequence counts per country from CoVariants^54^ and total tests per country from Worldometer^55^. Variables of interest were extracted from the original datasets and manually merged based on the “Country” identifier, in Microsoft Excel comma separated values (.csv) format.

**Table 5:** Data description, sources, and access.

Variable^a,b^	Dataset	Source	Access	Ref
Identifier				
Country	WHO-COVID-19-global-table-data	WHO	Public	^29^
Dependent				
CovDp100K	WHO-COVID-19-global-table-data	WHO	Public	^29^
Independent				
Geographic				
WHO_Region	WHO-COVID-19-global-table-data	WHO	Public	^29^
AvgTemp	Average temperature by country	Trading Economics	With permission	^56^
Demographic				
pPopFem^d^	Population, female (% of total population)	The World Bank	CC-BY 4.0	^57^
pPop65	Population ages 65 and above (% of total population)	The World Bank	CC-BY 4.0	^58^
pPopUrb	Urban population (% of total population)	The World Bank	CC-BY 4.0	^59^
AdLit	2022/04/2021-GHS-Index-April-2022	NTI/JHCHS/EI	Public	^60^
Socioeconomic				
GDPpC^c^	GDP per capita (current US$)	The World Bank	CC-BY 4.0	^61^
IGSpGDP^c^	Imports of goods and services (% of GDP)	The World Bank	CC-BY 4.0	^62^
pWfUem	Unemployment, total (% of total labor force) (modeled ILO estimate)	The World Bank	CC-BY 4.0	^63^
CPI	2022/04/2021-GHS-Index-April-2022	NTI/JHCHS/EI	Public	^60^
Healthcare				
HEpGDP	Current health expenditure (% of GDP)	The World Bank	CC-BY 4.0	^64^
GHS.In^c^	2022/04/2021-GHS-Index-April-2022	NTI/JHCHS/EI	Public	^60^
LEB	Life expectancy at birth, total (years)	The World Bank	CC-BY 4.0	^65^
HBp100K	2022/04/2021-GHS-Index-April-2022	NTI/JHCHS/EI	Public	^60^
MDp100K^d^	2022/04/2021-GHS-Index-April-2022	NTI/JHCHS/EI	Public	^60^
Population health				
NCDMortp100K^d^	2022/04/2021-GHS-Index-April-2022	NTI/JHCHS/EI	Public	^60^
pPopH_2_0^d^	People using at least basic drinking water services (% of population)	The World Bank	CC-BY 4.0	^66^
Pandemic				
PoeMgt	2022/04/2021-GHS-Index-April-2022	NTI/JHCHS/EI	Public	^60^
CovCp100K	WHO-COVID-19-global-table-data	WHO	Public	^29^
Delta21Jp100K	21J.Delta_table Reported cases and deaths by country or territory (total tests)	CoVariants Worldometer	CC-BY 4.0 Public	^54,55^
VacFullp100	who-data/vaccination-data	WHO	Public	^67^

AdLit = adult literacy rate (percent ≥ 15 years^27^); AvgTemp = average temperature (2021); CC-BY 4.0 = Creative Commons Attribution 4.0 International license; CovCp100K = COVID-19 Cases/100,000 population; CovDp100K = COVID-19 Deaths/100,000 population; CPI = Corruption Perception Index (0–100, where 100 = best^27^); Delta21Jp100K = COVID-19 Delta 21J sequence count/100,000 tests; EI = Economist Impact; GDP = gross domestic product; GDPpC = GDP per capita (US$); GHS = Global Health Security; GHS.In = Global Health Security Index (0–100, where 100 = best^27^); HBp100K = hospital beds/100,000 population; HEpGDP = health expenditure (% of GDP); IGSpGDP = imports of goods and services (% of GDP); JHCHS = Johns Hopkins Center for Health Security; LEB = life expectancy at birth; MDp100K = doctors/100,000 population; NCDMortp100K = non-communicable disease (age-standardized) mortality rate/100,000 population; NTI = Nuclear Threat Initiative; PoeMgt = point of entry management; pPop65 =  ≥ 65 years of age (percent of population); pPopFem = females (percent of population); pPopH_2_0 = using at least basic drinking water services (percent of population); pPopUrb = urban population (percent of population); Public = publicly available link/data; pWfUem = unemployment (percent of workforce); VacFullp100 = number of persons fully vaccinated/100 population; WHO = World Health Organization; WHO_Region = WHO region.

^a^Year of data also extracted with associated variable of interest values.

^b^Related independent variables grouped and analyzed as limited models.

^c^Variable removed due to high partial concurvity in limited model.

^d^Variable removed due to high partial concurvity in full model.

### Preprocessing

Raw data was read into R software with the *base*^22^ and *haven*^68^ packages, and comprised 221 cases (countries) with 41 variables, including country name, COVID-19 deaths, WHO region, average temperature, COVID-19 cases, Delta 21J sequence counts per country, total tests per country, Delta 21J sequence count/100,000 tests, and per capita vaccination, in addition to 16 other potential independent variables and their associated year (Table 5). The categorical variable PoeMgt, originally coded as 0, 1, and 2, but normalized to 0, 50, and 100 respectively, to make them directly comparable with other indicators^27^, was re-coded (*mutate* function from *dplyr* package^69^) to reflect the underlying categories as defined in the source documentation^27^. Data was screened for duplicates (*distinct* function from *dplyr* package^69^), invalid entries (empty rows or columns), and missing data (*is.na* function from R base package^22^). Countries with incomplete data were removed with the *complete.cases* function from the *stats* package^70^, and lack of missing values subsequently confirmed. Outliers, defined as datapoints > Q3 + 3 × IQR or < Q1 – 3 × IQR, were then detected with the *rstatix* package^71^. Finally, the *e1071* package^72^ was used to compute descriptive statistics.

### Statistical analysis

Due to non-linearity and outliers among continuous bivariate models, Spearman’s correlation (*correlation* package^73^) was used to examine the associations between COVID-19 mortality and continuous variables. The relationship with PoeMgt was tested with ANOVA (*stats* package^70^), but with WHO_Region using Welch’s Heteroscedastic F Test (*onewaytests* package^74^), because of heteroscedasticity between variable groups. Based on the presence of non-linear heteroscedastic multivariate models, country-level independent predictors of COVID-19 mortality were identified by weighted generalized additive models (GAMs) using the *gam* function (*mgcv* package:^75^). Due to cone-shaped residual plots, inverse error variances were applied as weights, as previously described for weighted least squares^76,77^. Briefly, model residuals were regressed on model fitted values, and weights estimated as the inverse of the squared extracted fitted values. GAMs apply smooth functions to continuous independent variables that capture non-linear aspects of non-linear relationships, with each flexible smooth comprised of smaller basis functions that model a portion of the relationship^78^. Adequacy of basis functions (model complexity) and concurvity were tested for all models. GAMs were fitted without adjusting model complexity (k-value), using a smoothing parameter of 0.0001 to minimize risk of overfitting^78^, and after removal of model variables with high partial concurvity (Table 5) defined as > 0.8 between a variable pair^28^. Non-significant or less significant high-concurvity variables, were removed first. Limited models were initially fitted for groups of related variables (Table 5), and then a final (full) model for all variables that did not violate the concurvity limit. Each model was evaluated with Cohen’s R^2^ and f^2^ to identify practically significant models defined as at least medium effect size: Cohen’s R^2^ > 13% or f^2^ > 0.15^23^. The impact of individual variables was then assessed with partial effects plots. All seven models were fitted with multiple linear regression, fitted with standardized independent variables, and the final model was run with various WHO regions as reference, for comparison. Trend changes for identified independent predictors were further assessed, by comparing the full model with the corresponding smooth term versus the linear term, using the F-test^25^. Differences in continuous independent predictors across WHO regions was also tested with Welch’s Heteroscedastic F Test (*onewaytests* package^74^. Finally, the full model was tested by backward elimination (R^2^ criterion) to determine the most parsimonious model. All data preprocessing, statistical analysis, and data visualization were performed with R, version 4.2.1 (The R Foundation for Statistical Computing, 2022). A p value of < 0.05 was considered statistically significant. However, for multiple comparisons, p-values were adjusted by Bonferroni correction.

## Supplementary Information

Supplementary Information 1.

Supplementary Information 2.

Supplementary Information 3.

Supplementary Information 4.

## Data Availability

The dataset used in this paper was compiled from publicly available sources, each with or without a specified licence, as outlined in Table 5. The sources include two datasets from the World Health Organization; one from Trading Economics; nine from The World Bank; and one each from the Nuclear Threat Initiative/Johns Hopkins Center for Health Security/Economist Impact, CoVariants, and Worldometer, as follows: (1) WHO-COVID-19-global-table-data. WHO [accessed December 16th 2022]: https://covid19.who.int/WHO-COVID-19-global-table-data.csv. (2) WHO-data/vaccination-data. WHO [accessed December 13th 2022]: https://covid19.who.int/who-data/vaccination-data.csv. (3) Average Temperature by Country. Trading Economics [accessed December 5th 2022]: https://tradingeconomics.com/country-list/temperature. (4) Population, female (% of total population). The World Bank [accessed November 7th 2022]: https://data.worldbank.org/indicator/SP.POP.TOTL.FE.ZS. (5) Population ages 65 and above (% of total population). The World Bank [accessed November 7th 2022]: https://data.worldbank.org/indicator/SP.POP.65UP.TO.ZS. (6) Urban population (% of total population). The World Bank [accessed November 7th 2022]: https://data.worldbank.org/indicator/SP.URB.TOTL.IN.ZS. (7) GDP per capita (current US$). The World Bank [accessed October 15th 2022]: https://data.worldbank.org/indicator/NY.GDP.PCAP.CD. (8) Imports of goods and services (% of GDP). The World Bank [accessed November 7th 2022]: https://data.worldbank.org/indicator/NE.IMP.GNFS.ZS. (9) Unemployment, total (% of total labor force) (modeled ILO estimate). The World Bank [accessed November 7th 2022]: https://data.worldbank.org/indicator/SL.UEM.TOTL.ZS. (10) Current health expenditure (% of GDP). The World Bank [accessed October 15th 2022]: https://data.worldbank.org/indicator/SH.XPD.CHEX.GD.ZS. (11) Life expectancy at birth, total (years). The World Bank [accessed November 7th 2022]: https://data.worldbank.org/indicator/SP.DYN.LE00.IN. (12) People using at least basic drinking water services (% of population). The World Bank [accessed November 7th 2022]: https://data.worldbank.org/indicator/SH.H2O.BASW.ZS. (13) 2022/04/2021-GHS-Index-April-2022. Nuclear Threat Initiative/Johns Hopkins Center for Health Security/Economist Impact [accessed October 31st 2022]: https://www.ghsindex.org/wp-content/uploads/2022/04/2021-GHS-Index-April-2022.csv. (14) CoVariants: SARS-CoV-2 Mutations and Variants of Interest. CoVariants [accessed December 15th 2022]: https://github.com/hodcroftlab/covariants/blob/83d7fdf5a782193ef64d82d8ddd93cdbfa889539/cluster_tables/21J.Delta_table.tsv. (15) Reported Cases and Deaths by Country or Territory. Worldometer [accessed December 16th 2022]: https://www.worldometers.info/coronavirus/. The compiled raw data is available as a supplementary file.

## Code availability

R scripts used for data preprocessing, statistical analysis, and data visualization are available as supplementary files.
