# Supplementary material for: Country-level predictors of COVID-19 mortality
Source: Sci Rep. 2023 Jun 7;13:9263. doi: 10.1038/s41598-023-36449-x (PMC10245344; doi:10.1038/s41598-023-36449-x)
Supplement: Supplementary file 2 — Supplementary Information 2. [file 41598_2023_36449_MOESM2_ESM.pdf]

Country-level predictors of COVID-19 mortality

Paul A Brown*

Supplementary Table S1 Pairwise comparisons of mean COVID-19 deaths across WHO regional groups

Level (a)	Level (b)	p value	Null hypothesis
Africa	Americas	0.000	Reject
Africa	Eastern Mediterranean	0.196	Not reject
Africa	Europe	0.000	Reject
Africa	South-East Asia	0.694	Not reject
Africa	Western Pacific	1.000	Not reject
Americas	Eastern Mediterranean	0.001	Reject
Americas	Europe	1.000	Not reject
Americas	South-East Asia	0.000	Reject
Americas	Western Pacific	0.000	Reject
Eastern Mediterranean	Europe	0.000	Reject
Eastern Mediterranean	South-East Asia	1.000	Not reject
Eastern Mediterranean	Western Pacific	1.000	Not reject
Europe	South-East Asia	0.000	Reject
Europe	Western Pacific	0.000	Reject
South-East Asia	Western Pacific	1.000	Not reject

[a] Welch's Heteroscedastic F Test: Bonferroni Correction (alpha = 0.05)

Supplementary Table S2 Pairwise comparisons of mean percent of population ≥ 65 years of age across WHO regional groups

Level (a)	Level (b)	p value	Null hypothesis
Africa	Americas	0.000	Reject
Africa	Eastern Mediterranean	1.000	Not reject
Africa	Europe	0.000	Reject
Africa	South-East Asia	0.189	Not reject
Africa	Western Pacific	0.068	Not reject
Americas	Eastern Mediterranean	0.000	Reject
Americas	Europe	0.000	Reject
Americas	South-East Asia	1.000	Not reject
Americas	Western Pacific	1.000	Not reject
Eastern Mediterranean	Europe	0.000	Reject
Eastern Mediterranean	South-East Asia	0.745	Not reject
Eastern Mediterranean	Western Pacific	0.203	Not reject
Europe	South-East Asia	0.000	Reject
Europe	Western Pacific	0.063	Not reject
South-East Asia	Western Pacific	1.000	Not reject

[a] Welch's Heteroscedastic F Test: Bonferroni Correction (alpha = 0.05)

Supplementary Table S3 Pairwise comparisons of mean Corruption Perception Index across WHO regional groups

Level (a)	Level (b)	p value	Null hypothesis
Africa	Americas	0.471	Not reject
Africa	Eastern Mediterranean	1.000	Not reject
Africa	Europe	0.000	Reject
Africa	South-East Asia	1.000	Not reject
Africa	Western Pacific	0.238	Not reject
Americas	Eastern Mediterranean	1.000	Not reject
Americas	Europe	0.023	Reject
Americas	South-East Asia	1.000	Not reject

Americas	Western Pacific	1.000	Not reject
Eastern Mediterranean	Europe	0.004	Reject
Eastern Mediterranean	South-East Asia	1.000	Not reject
Eastern Mediterranean	Western Pacific	1.000	Not reject
Europe	South-East Asia	0.000	Reject
Europe	Western Pacific	1.000	Not reject
South-East Asia	Western Pacific	0.387	Not reject

[a] Welch's Heteroscedastic F Test: Bonferroni Correction (alpha = 0.05)

Supplementary Table S4 Pairwise comparisons of mean hospital beds/100,000 population across WHO regional groups

Level (a)	Level (b)	p value	Null hypothesis
Africa	Americas	0.571	Not reject
Africa	Eastern Mediterranean	1.000	Not reject
Africa	Europe	0.000	Reject
Africa	South-East Asia	1.000	Not reject
Africa	Western Pacific	0.264	Not reject
Americas	Eastern Mediterranean	1.000	Not reject
Americas	Europe	0.000	Reject
Americas	South-East Asia	1.000	Not reject
Americas	Western Pacific	0.870	Not reject
Eastern Mediterranean	Europe	0.000	Reject
Eastern Mediterranean	South-East Asia	1.000	Not reject
Eastern Mediterranean	Western Pacific	0.501	Not reject
Europe	South-East Asia	0.009	Reject
Europe	Western Pacific	1.000	Not reject
South-East Asia	Western Pacific	0.933	Not reject

[a] Welch's Heteroscedastic F Test: Bonferroni Correction (alpha = 0.05)

Supplementary Table S5 Pairwise comparisons of mean COVID-19 cases/100,000 population across WHO regional groups

Level (a)	Level (b)	p value	Null hypothesis
Africa	Americas	0.000	Reject
Africa	Eastern Mediterranean	0.150	Not reject
Africa	Europe	0.000	Reject
Africa	South-East Asia	1.000	Not reject
Africa	Western Pacific	0.095	Not reject
Americas	Eastern Mediterranean	1.000	Not reject
Americas	Europe	0.000	Reject
Americas	South-East Asia	1.000	Not reject
Americas	Western Pacific	1.000	Not reject
Eastern Mediterranean	Europe	0.000	Reject
Eastern Mediterranean	South-East Asia	1.000	Not reject
Eastern Mediterranean	Western Pacific	1.000	Not reject
Europe	South-East Asia	0.001	Reject
Europe	Western Pacific	0.290	Not reject
South-East Asia	Western Pacific	1.000	Not reject

[a] Welch's Heteroscedastic F Test: Bonferroni Correction (alpha = 0.05)

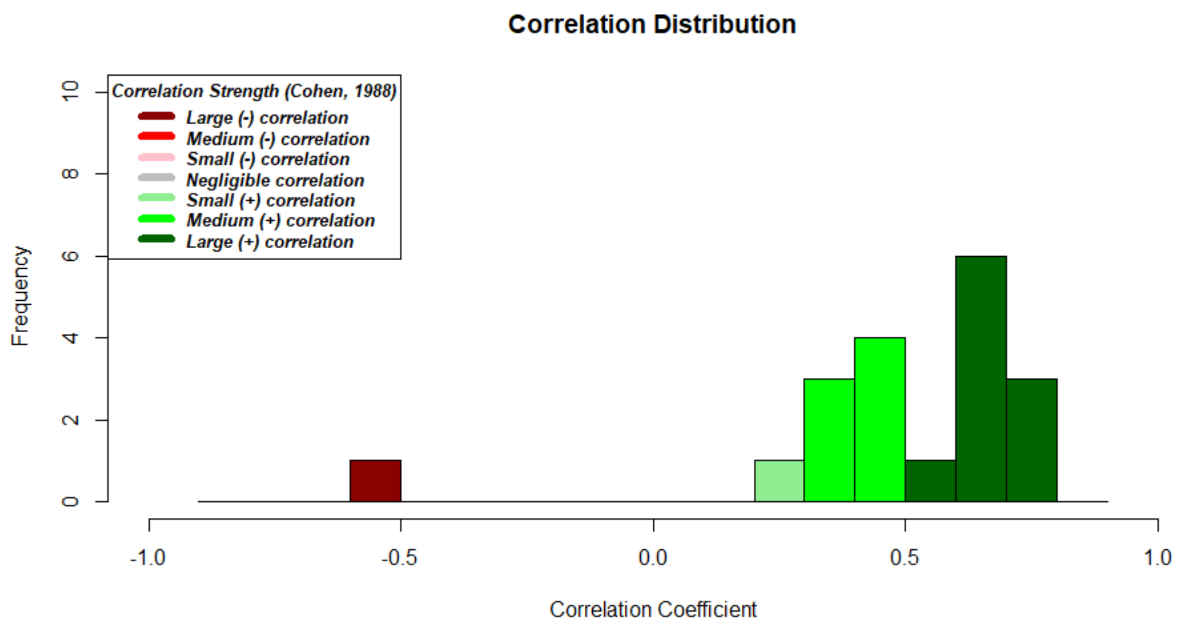

Supplementary Figure S1 Distribution of correlations

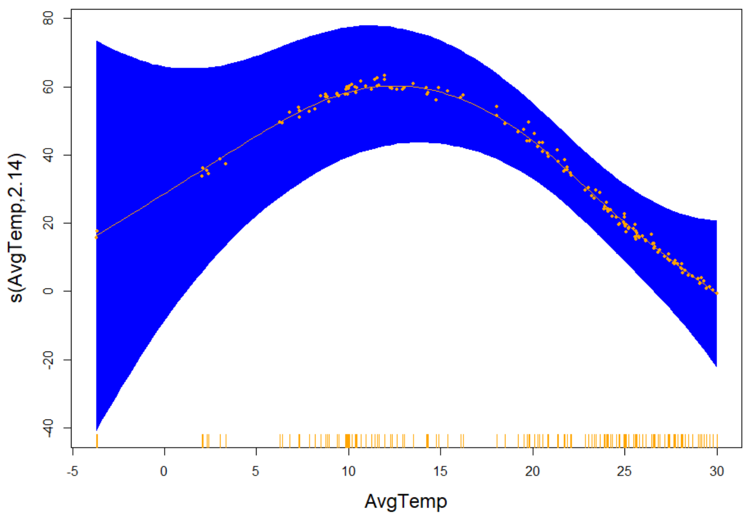

Supplementary Figure S2 Partial effects plot for gam.MODEL_1Geo model

Plotted with the *shift* argument to shift the scale based on the intercept value, for a more natural interpretation [1]. The smooth function on y-axis therefore represents the partial effect of the independent variable on COVID-19 mortality (presented as the independent variable, with effective degrees of freedom). Shading reflects 95% confidence interval for the mean shape of the effect [1].

AvgTemp = average temperature (2021).

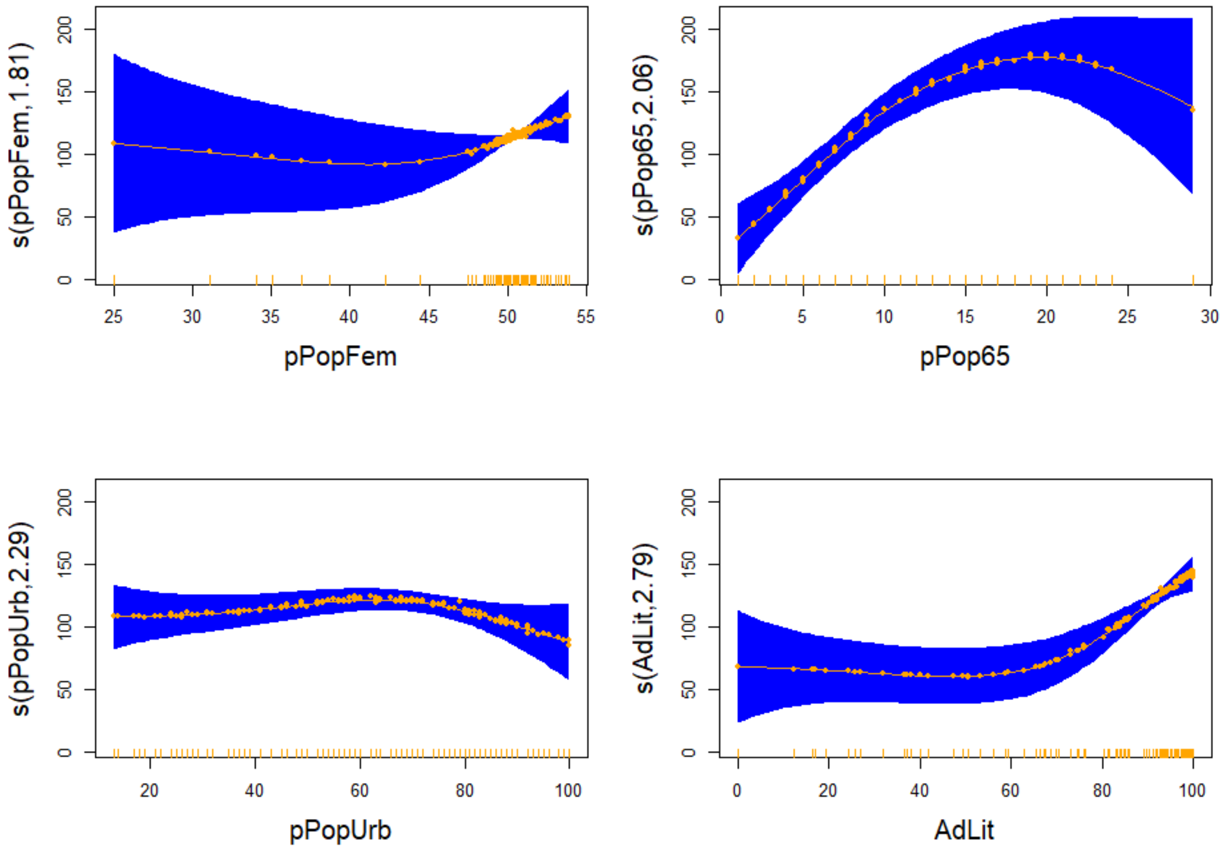

Supplementary Figure S3 Partial effects plots for gam.MODEL_2Dem model

Plotted with the *shift* argument to shift the scale based on the intercept value, for a more natural interpretation [1]. The smooth function on y-axis therefore represents the partial effect of the independent variable on COVID-19 mortality (presented as the independent variable, with effective degrees of freedom). Shading reflects 95% confidence interval for the mean shape of the effect [1].

pPopFem = females (percent of population); pPop65 = ≥ 65 years of age (percent of population); pPopUrb = urban population (percent of population); AdLit = adult literacy rate (percent ≥ 15 years).

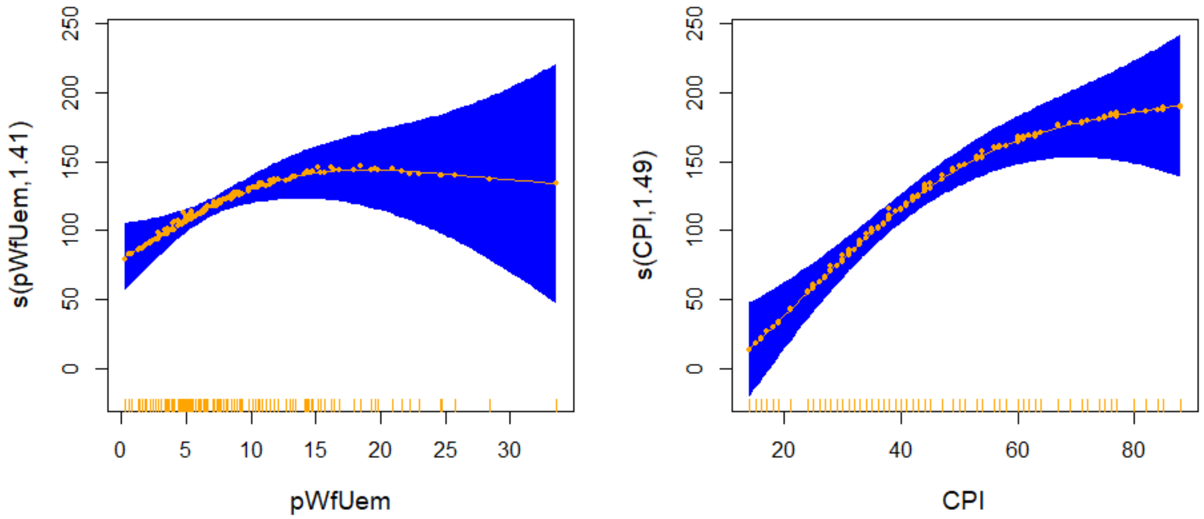

Supplementary Figure S4 Partial effects plots for gam.MODEL_3SoEc_mod model

Plotted with the *shift* argument to shift the scale based on the intercept value, for a more natural interpretation [1]. The smooth function on y-axis therefore represents the partial effect of the independent variable on COVID-19 mortality (presented as the independent variable, with effective degrees of freedom). Shading reflects 95% confidence interval for the mean shape of the effect [1].

pWfUem = unemployment (percent of workforce); CPI = Corruption Perception Index.

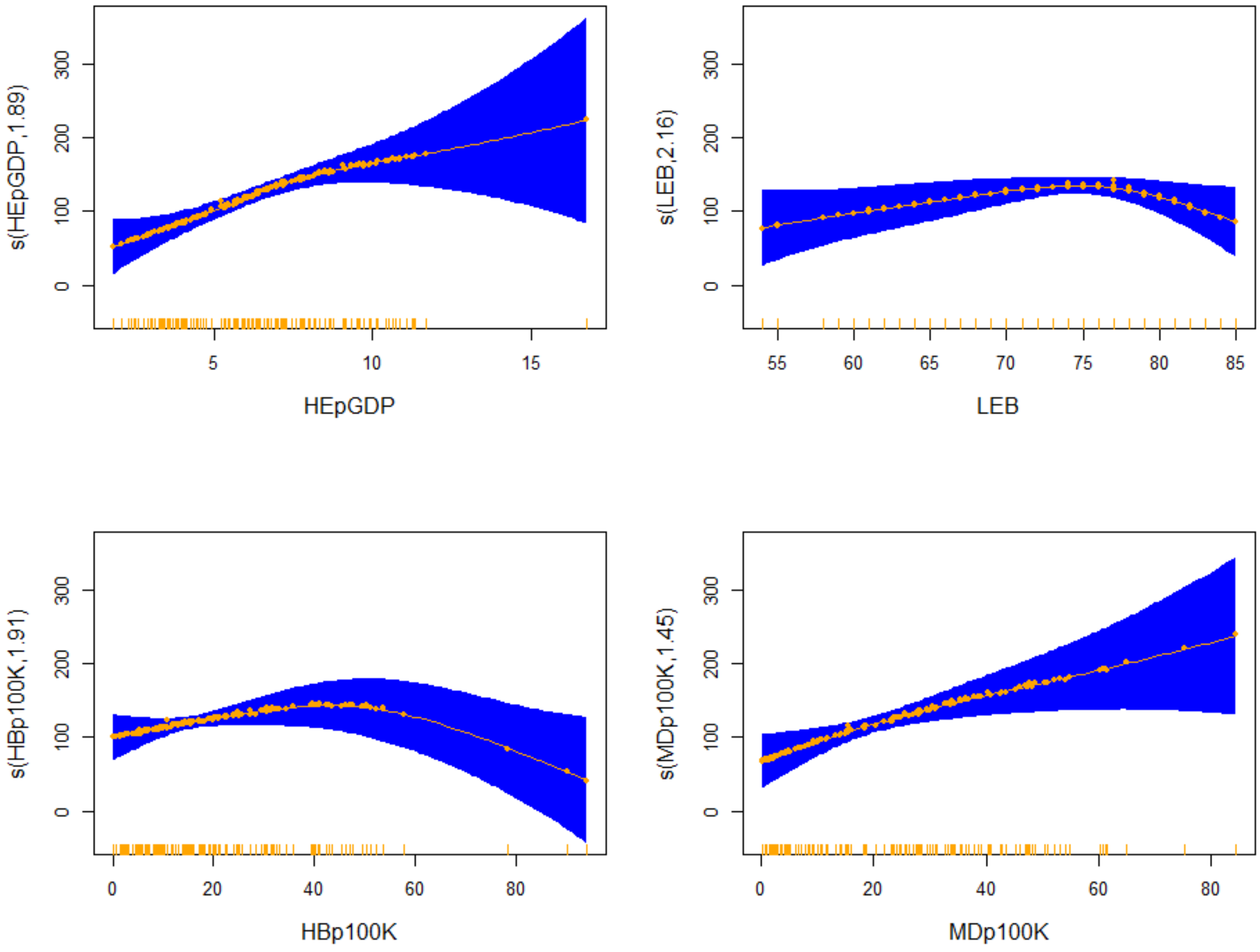

Supplementary Figure S5 Partial effects plots for gam.MODEL_4Heal_mod model

Plotted with the *shift* argument to shift the scale based on the intercept value, for a more natural interpretation [1]. The smooth function on y-axis therefore represents the partial effect of the independent variable on COVID-19 mortality (presented as the independent variable, with effective degrees of freedom). Shading reflects 95% confidence interval for the mean shape of the effect [1].

HEpGDP = health expenditure (% of GDP); LEB = life expectancy at birth; HBp100K = hospital beds/100,000 population; MDp100K = doctors/100,000 population.

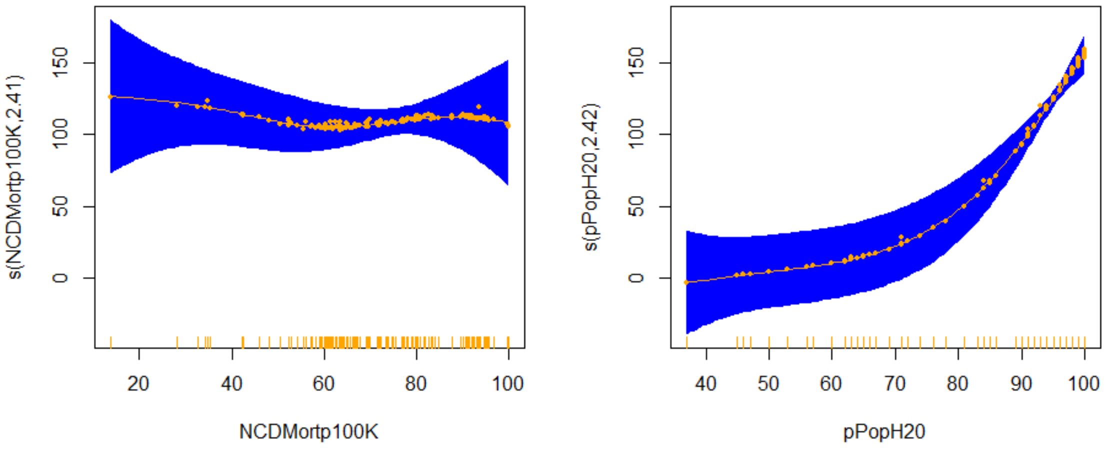

Supplementary Figure S6 Partial effects plots for gam.MODEL_5PopH model

Plotted with the *shift* argument to shift the scale based on the intercept value, for a more natural interpretation [1]. The smooth function on y-axis therefore represents the partial effect of the independent variable on COVID-19 mortality (presented as the independent variable, with effective degrees of freedom). Shading reflects 95% confidence interval for the mean shape of the effect [1].

NCDMortp100K = non-communicable disease (age-standardized) mortality rate/100,000 population; pPopH20 = using at least basic drinking water services (percent of population).

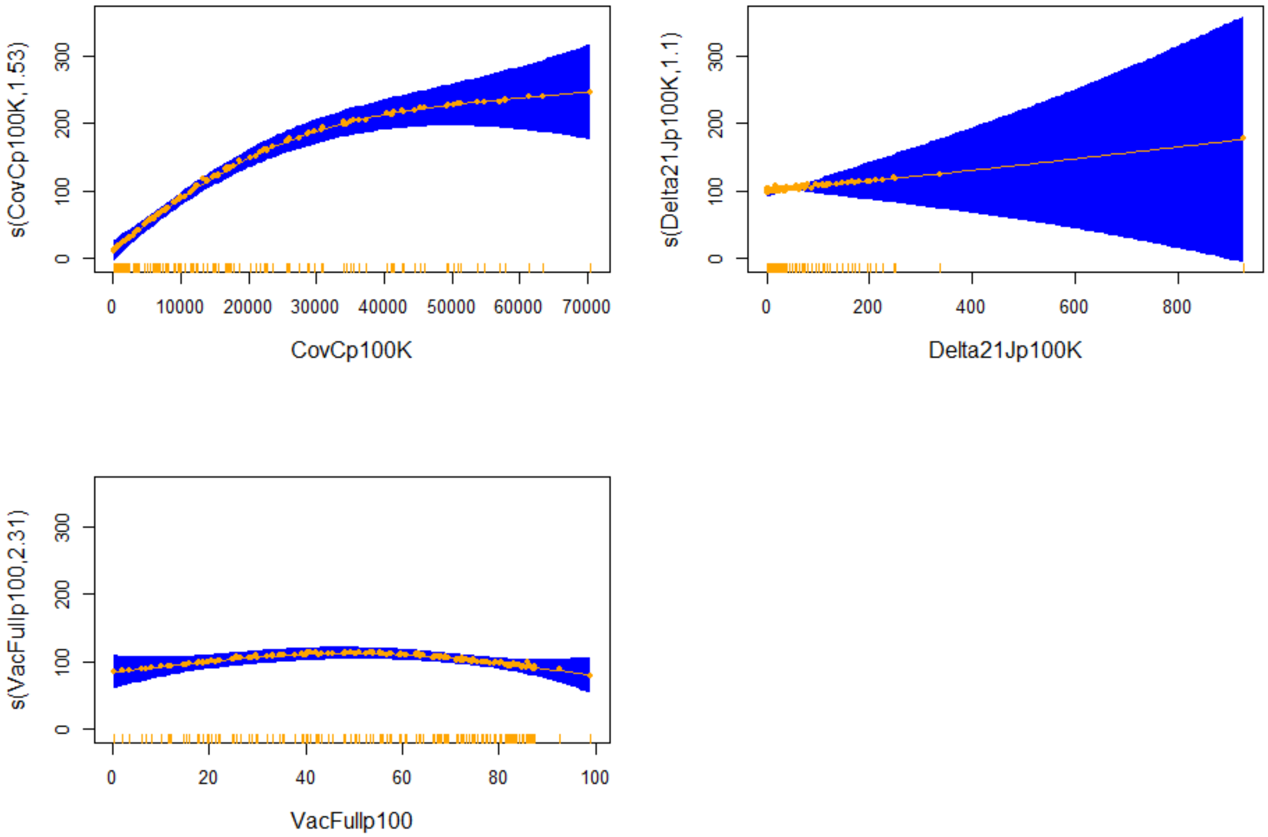

Supplementary Figure S7 Partial effects plots for gam.MODEL_6Pand model

Plotted with the *shift* argument to shift the scale based on the intercept value, for a more natural interpretation [1]. The smooth function on y-axis therefore represents the partial effect of the independent variable on COVID-19 mortality (presented as the independent variable, with effective degrees of freedom). Shading reflects 95% confidence interval for the mean shape of the effect [1].

CovCp100K = COVID-19 Cases/100,000 population; Delta21Jp100K = COVID-19 Delta 21J sequence count/100,000 tests; VacFullp100 = number of persons fully vaccinated/100 population.

REFERENCES

1 Ross, N., Miller, D., Simpson, G. L. & Pedersen, E. J. *Generalized Additive Models in R. Chapter 2.*, <https://noamross.github.io/gams-in-r-course/chapter2 (Accessed 03.04.2022)> (2019).
